# Immunodiagnostic Methods: What Is Their Role in Areas of Low Endemicity?

**DOI:** 10.1100/2012/593947

**Published:** 2012-12-17

**Authors:** Rafaella Fortini Queiroz Grenfell, Vanessa Silva-Moraes, Diana Taboada, Ana Carolina Alves de Mattos, Ana Karine Sarvel de Castro, Paulo Marcos Zech Coelho

**Affiliations:** Schistosomiasis Laboratory, Rene Rachou Research Center, Oswaldo Cruz Foundation (Fiocruz), Avenida Augusto de Lima, 1715/201 Belo Horizonte, MG, Brazil

## Abstract

Worldwide *Schistosomiasis mansoni* continues to be a serious public health problem. Over the past decades, control programmes have made remarkable progress in reducing *S. mansoni* infections to a relatively low level in Brazil and African countries. Endemic regions are currently circumscribed in certain core areas where reinfection and repeated chemotherapy are frequent and, consequently, are related to residents with low parasite load. At present, diagnosis is predominately a key step for final disease control although low endemicity area residents are hardly detected by most of the available assays. In this paper, we review the current status and efforts made aiming at the improvement of diagnostic tools for *S. mansoni* in low endemicity infections. The establishment of diagnostic assays—simple, affordable, sensitive, and specific for field diagnosis of *S. mansoni*—is essential and should be given high priority.

## 1. Introduction

Schistosomiasis is a complex of acute and mainly chronic diseases caused by six *Schistosoma* species: *Schistosoma haematobium*, *S. guineensis*, *S. intercalatum*, *S. mansoni*, *S. japonicum,* and *S. mekongi* [1]. Schistosomiasis occurs in the tropics and subtropics and is among the most important parasitic diseases worldwide, with a significant socioeconomic impact [2]. Seventy-four countries are endemic, with roughly 120 million individuals being symptomatically infected and 20 million being severely affected [3]. Moreover, schistosomiasis represents an increasing problem in nonendemic areas, due to the growing number of immigrants and tourists [4–6]. The acute symptomatic schistosomiasis may appear 3 to 7 weeks after an exposure and is characterized by cough, fever, anorexia, abdominal pain, headaches, and hypereosinophilia when the inflammatory reaction is well established involving predominantly Th1 biased and elevated immunoglobulin levels in sera [7]. The late acute phase that occurs after 40 to 90 days following infection is characterized by the egg laying and a predominantly Th2 inflammatory response. Its clinical manifestations vary depending on the parasite load and host immune response when chronic phase is established.

 In Brazil the elimination of the disease is considered but there are areas of low prevalence where elimination could be achieved with adequate resources and political commitment. In some countries, elimination of schistosomiasis is already the declared goal. Hence, although morbidity control is likely to remain the main strategy in most places, it is essential to ensure that adequate tools for transmission control and innovative surveillance-response mechanisms are in place wherever elimination is to be achieved [8].

Since praziquantel was developed in 1970s, it has replaced other antischistosomal drugs to become the only drug of choice for treatment of human schistosomiasis, due to high efficacy, excellent tolerability, few and transient side effects, simple administration, and competitive cost [9]. Lessons learnt from the successes and failures of past control and elimination programmes must not be ignored. Emphasis on the large-scale administration of praziquantel not combined to the individual diagnosis was largely adopted during the 1970s as part of the Special Program for Schistosomiasis Control in Brazil, and it is still in progress in Africa [8]. Although this strategy could have led to an effective control of parasite transmission, it showed a different scenario in endemic areas. There is evidence that prevalence and worm burden have decreased significantly since the introduction of this program [10–12]. Given this fact, new objectives need to be defined transferring from control programs designed for morbidity reduction to strategies for transmission control [13].

In a recent review [9] the authors show the absence of exact mechanisms of action of praziquantel, the mechanisms of drug resistance in schistosomes that remain unclear. The study evidences the urgent and great importance to strengthen the monitoring of praziquantel sensitivity and detection of praziquantel resistance in schistosomes and a development of an effective and rapid detection technique that is urgently needed.

Prevalence and egg burden in feces is expected to be low in areas either under advanced control of transmission [14] or with recent introduction [15, 16]. The increasing number of individuals showing low parasite load, most of the time with less than 10 eggs per gram of feces after extensive examination, turns the diagnosis even harder to be performed. Moreover, the exact definition of the prevalence of a population, as well as the identification of asymptomatic carriers, becomes unviable because a large amount of unnecessary stool examinations has to be carried out to find a relatively small number of infected individuals.

Herein, diagnosis plays a crucial role in the monitoring of the infection as well as efficacy of treatment. Currently, the “gold” standard remains the detection of schistosome eggs in stools. The Kato-Katz technique is the most widely used copromicroscopic method in epidemiological surveys [17]. However, because of low egg production in endemic areas, the risk of having a large number of individuals who remain undiagnosed is considerable. Consequently, undiagnosed individuals remain infected and contribute to transmission of the disease. Siqueira et al. [18] showed that the prevalence obtained from the examination of one Kato-Katz slide in a low-transmission area in Brazil (the methodology adopted by the Brazilian control programme) was 8% compared to 35.8% from the “gold standard” (Kato-Katz method—18 slides plus a quantitative commercial test—TF-Test), which was a 4.5-fold difference.

Development and implementation of optimal methodologies for diagnosis is crucial in all aspects of schistosomiasis control, and high sensitivity, as well as absolute specificity, will be needed as programmes shift their emphasis from control to elimination [19]. Since initial disease surveillance must be enhanced so that drug delivery can be focused [20], research has been mainly focused on the development and optimization of improved diagnostic methods in order to achieve significant diagnosis sensitivity for the detection of infections with low parasite load.

## 2. Immunodiagnostic Methods for Antibodies Detection

New indirect diagnostic tests based on antibody detection have been developed. Indirect methodologies are highly sensitive even when parasite burden is low, but they are unable to distinguish past from current infections and are relatively nonspecific and prone to cross-reactions with other antigens [5, 21]. Since it is based on the determination of the humoral immune response, positive antibody test results may take an indefinite period of time to convert to negativity after elimination of infection [22] and do not reflect the intensity of infection [23].

The presence of cross-reactive IgG antibodies in serum samples of schistosomiasis patients was detected by different authors when tested with antigens of other parasite species by ELISA and Western blot [24–26]. Nevertheless, good specificity (98.2%) was observed when detection of IgM antibodies to gut-associated antigens by immunofluorescent test (IFT). This IFT was applied in different areas of the State of São Paulo, Brazil, with no schistosomiasis and high prevalence for other helminth infections, such as ascaridiasis, trichuriasis, and enterobiasis [27]. There are various studies that assess the potential of different immunodiagnostic methods in populations [28–31], and, even when applied in low endemicity areas, these immunodiagnostic assays show a good relation between sensitivity and positivity ratio but still cannot discriminate the active infection.

Nonetheless, these methodologies have their own advantages when leading with low endemicity infections as confirmatory diagnosis. That said, schistosome egg antigen (SEA) enzyme-linked immunosorbent assay (ELISA) can be used as a complementary field-based method for monitoring infection. It performs well over a range of endemic settings and would be best applied to monitor the incidence of “new” infections in young children in environments where transmission was thought to be interrupted [21]. The most important point to be considered is that the use of different antigens in immunological methods can be applied as potential tools for the analysis of the chronological evolution of *S. mansoni* infection [32]. Recent analysis has shown that the antigens from different life stages as schistosomula, adult worms, and eggs can be markers for prepatent, acute and chronic phases. Results from individual laboratories and, from multicentre trials, suggest that egg antigens provide greater diagnostic sensitivity and specificity than worm antigens for the detection of an infection [33, 34].

It is clear however that diagnostic tests based on egg antigens should be postponed until egg laying is started. To obtain a positive result, the parasite cycle must be completed within the definitive host with the development of male and female adult worms, which reproduce and lead to the oviposition [35]. Also, although extracts prepared by homogenizing *Schistosoma *eggs contain a large number of molecules, only a minority of the constituents of egg antigen might be released by viable eggs *in vivo*, as demonstrated *in vitro *[36], which can explain the low detection capability of an SEA-ELISA assay.

On the contrary, others have shown that during acute phase of the disease there is an increase in anti-worm antibody titers, and this fact may be due to the production of antibodies specific for glycan epitopes that schistosome larvae and adult worms [37] have in common. Since 2004 there has been a remarkable increment in proteomics, especially for proteins that reside at the surfaces of schistosome in the mammalian host. The most important proteins secreted by the cercaria to gain access to the skin have been described as well as those from schistosomula [38]. At this manner, schistosomula tegument antigens have been considered as important markers for the diagnosis and, although the identification of tegument proteins has been the first step [39–41], it is the glycans that have been real evidence. The detailed identification of glycans on the surface of all schistosome life stages has announced that some glycans are preserved on the tegument during all the parasite life, and most importantly some of the most immunogenic glycans are exclusively found in *Schistosoma *sp. [42]. The application of these glycans to innovative immunodiagnostic methods has now been strongly recommended.

## 3. Immunodiagnostic Methods for Circulating Antigens Detection

 Besides indirect immunoassays, the diagnosis of schistosomiasis was increasingly improved by the development of new methods aiming at the detection of circulating anodic and cathodic antigens (CAA and CCA) in blood or urine [43]. CCA is regurgitated from worms into the circulatory system and later is eliminated in urine. Since CCAs are only released from living worms, the rapid tests can be used to monitor the dynamics of existing worm burdens, as well as clearance following treatment [43, 44]. However, these assays were initially presented as cumbersome and with a low rate of sensitivity even for the diagnosis of patients with high parasite load. Current studies has confirmed that improvements in the initial methodology allowed the validation of a new method for the diagnosis of active infection presenting very high sensitivity and specificity for the detection of hard-to-detect patients. Additionally, rapid diagnostic tests detecting CCA of *S. mansoni *are also readily available in dipstick or cassette format. Recent studies carried out in different epidemiological settings of Côte d'Ivoire and Kenya revealed that a single CCA performed on urine samples shows equal or even higher sensitivity for *S. mansoni *diagnosis than multiple Kato-Katz thick smears obtained from stool samples [45, 46].

For utilization under field conditions, an assay should be rapid, specific and, most importantly, sensitive enough to discriminate between active infections. That is why fluorescence imaging has also become a valuable approach for antigen detection [47]. This method may be more cost-effective as well as more accurate, rapid and easy-to-do than quantitative ones. On the other hand, it depends on the technicians' observation, and differences on the final data could be seen for different technicians.

## 4. Conclusion

We are currently in a diagnostic dilemma for *S. mansoni*—the direct parasitological major technique (Kato-Katz) have become relatively insensitive due to widespread chemotherapy that results in generally low worm burdens, which leads to less efficiency in low transmission settings and in post-treatment situations [2, 8, 10, 14, 25, 27, 30, 37]. Other diagnostic alternatives include detection of antibodies or circulating antigens in serum and/or urine samples. Antibody detection assays with relatively high sensitivity but generally low specificity, do not differentiate between current and cured infections, which results in the difficulties in determining prevalence, identifying true infected individuals for selective chemotherapy and assessing the effectiveness of intervention including follow-up of chemotherapy. The detection of circulating antigens was turned out to be a highly efficient and easy-to-do assay showing same or superior results than parasitological assays. Although extra validation is needed for CCA detection, these qualitative and/or quantitative methods showed far superior results than available immunodiagnostic assays [20, 21, 28, 43–46].

The diagnosis of schistosomiasis relies on microscopic examination of stools or urine, serologic tests, and imaging. However, taking into account the unsatisfactory diagnostic value of most of them in areas with low infection intensity, a search for a better diagnostic test that can be applied in field situations in Brazil and Africa should be given high priority. The role of immunodiagnosis in low endemicity areas has been repeatedly reaffirmed. The focus now relies on the improvement of sensitivity and specificity using new proteomics and, most importantly, glycomics recent information on antibody detection methods aiming innovative confirmatory diagnosis. Nonetheless, immunodiagnostic methods for circulating antigens level determination should be better validated in order to be able to establish individual parasite load as the primary diagnosis.
